# Rotational thromboelastometry and multiple electrode platelet aggregometry in four patients with abnormal routine coagulation studies before removal of epidural catheters after major surgery: a case series and research study

**DOI:** 10.1186/1752-1947-7-282

**Published:** 2013-12-30

**Authors:** Owain D Thomas, Anna Gustafsson, Ulf Schött

**Affiliations:** 1 Department of Anaesthesia and Intensive Care, Skåne University Hospital, S-22185 Lund, Sweden; 2 Faculty of Medicine, University of Lund, Lund, Sweden; 3 Uddevalla Hospital, Uddevalla, Sweden

**Keywords:** aPTT, Epidural anesthesia, Epidural hematoma, Hydroxyethyl starch, Multiplate®, Platelet aggregometry, PT-INR, ROTEM®, Thromboelastography

## Abstract

**Introduction:**

Routine coagulation tests have a low predictability for perioperative bleeding complications, and spinal hematoma after removal of epidural catheters is very infrequent. Thromboelastometry and point-of-care platelet aggregometry may improve hemostatic monitoring but have not been studied in the context of safety around epidural removal.

**Methods:**

Twenty patients who received an epidural catheter for major thoracoabdominal and abdominal surgery were included prospectively. In addition to routine coagulation tests, rotational thromboelastometry and multiple electrode platelet aggregometry were carried out.

**Results:**

A coagulation deficit was suggested by routine coagulation tests on the intended day of epidural catheter removal in four out of 20 patients. Prothrombin time-international normalized ratio was elevated to 1.5 in one patient (normal range: 0.9 to 1.2) while rotational thromboelastometry and multiple electrode platelet aggregometry parameters were within normal limits. Activated partial thromboplastin time was elevated to 47 to 50 seconds in the remaining three patients (normal range 28 to 45 seconds). Rotational thromboelastometry showed that one of the patients’ results was due to heparin effect: the clotting time with the HEPTEM® activator was 154 seconds as compared to 261 seconds with INTEM. The three remaining patients with prolonged routine coagulation test results had all received over 1L of hydroxyethyl starch (Venofundin®) and thrombosis prophylaxis with low-molecular-weight heparin (enoxaparin). Rotational thromboelastometry and multiple electrode platelet aggregometrygave normal or hypercoagulative signals in most patients.

**Conclusions:**

This case series is new in that it examines rotational thromboelastometry and multiple electrode platelet aggregometry postoperatively in the context of epidural analgesia and shows that they may be clinically useful. These methods should be validated before they can be used for standard patient care.

## Introduction

Analgesia and anesthesia by administration of a local anesthetic and an opiate through an epidural catheter provide effective pain control during and after major surgery, and are routinely used at our hospital. Hematoma within the spinal canal is a serious complication of epidural analgesia causing neurological damage and requiring urgent surgical decompression. They are most likely at the time of epidural catheterization, when the risk is estimated to be between 1:4000 and 1:30,000, and at the time of removal or manipulation of epidural catheters when the risk is estimated to be between 1:150,000 and 1:190,000 [1,2]. Patients who have undergone major surgery often have a coagulation deficit which may be caused by loss of coagulation factors and platelets due to surgical hemorrhage, preoperative malnutrition, systemic inflammation response syndrome, or due to accumulation or overdosing of thrombosis prophylaxis.

It is uncontroversial that preoperative coagulation deficits predispose to spinal hematoma at the time of epidural catheterization, but the sensitivity and specificity of routine coagulation tests, usually the prothrombin time-international normalized ratio (PT-INR), activated partial thromboplastin time (aPTT) and platelet count (Plc), in this context are unknown. These tests’ usefulness is questionable in patients who lack risk factors for perioperative bleeding, such as a history of bleeding or taking anticoagulant drugs [3-5]. It is more uncertain whether these tests can indicate the risk of hemorrhagic complications related to postoperative manipulation and removal of epidural catheters. A number of case reports suggest that point-of-care tests measuring whole blood viscoelasticity (e.g. thromboelastography (TEG®) and rotational thromboelastometry (ROTEM®)) and platelet aggregometry (e.g. multiple electrode platelet aggregometry (Multiplate®) and VerifyNow®) may be of use in regional anesthesia but evidence here is scarce.

Ahead of a larger study which is currently in progress, we carried out a pilot study, approved by The Swedish Central Ethical Review Board (Lund, DNR 2010/482). Signed consent was given by 20 consecutive patients who had an epidural catheter in place for analgesia after major gastrointestinal surgery. Our aim was to compare results from point-of-care and routine coagulation tests, hypothesizing that the whole blood assays ROTEM® and Multiplate® might give normal results despite moderately abnormal routine test results, which are run on plasma. This would of course be of interest since it is a common clinical scenario to be presented with a patient whose epidural catheter needs to be removed but whose routine coagulation parameters suggest a mild bleeding diathesis. We also compared preoperative routine coagulation results with postoperative results to confirm our clinical impression that the normal pattern of coagulation in these patients is a tendency towards coagulopathy as measured by PT-INR and aPTT.

## Results

Included in the study were 20 patients with a thoracic epidural catheter in place, 13 men and seven women. Of the 20 patients, 15 had undergone major gastrointestinal surgery by laparotomy alone and the other five had also undergone thoracotomy. The mean age was 58 years (range 26 to 83). None were treated with platelet inhibitors. Mean blood loss during their operation was 415mL (standard deviation 315mL). All patients received 500mL or more of synthetic colloid as hydroxyethyl starch 130/0.42 (Venofundin®). All were treated with thrombosis prophylaxis in a standard once daily dose at 8 p.m. of 40mg enoxaparin irrespective of weight. The mean time between epidural catheterization and removal was 5.6 days (range 2 to 14 days, standard deviation 2.8 days). A small number of routine test results were missing (four preoperative PT-INR values, one preoperative Plc and one postoperative aPTT).

### Routine test results in the 20 patients

**Figure 1:**
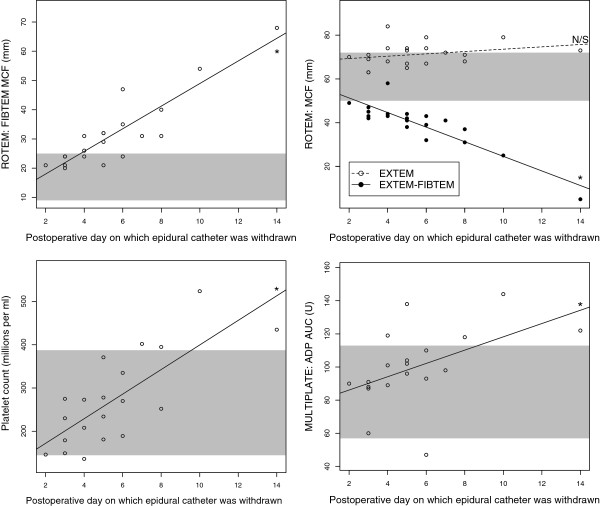
**Results from assays related mainly to platelet count and function.** These results give an overall impression of normo- or hypercoagulability. Shaded areas indicate the normal ranges. Platelet count, ROTEM®-FIBTEM®-maximum clot firmness (taken to be a quantitative measure of blood fibrinogen concentration) and Multiplate® adenosine diphosphate-area under curve (a measure of platelet activity) all correlated significantly to the length of time after surgery (p<0.05, r=0.89, 0.76 and 0.49 respectively). ROTEM®-EXTEM-maximum clot firmness, an overall measure of the extrinsic pathway, neither increased nor decreased with time after operation while ROTEM®-(EXTEM minus FIBTEM®)-maximum clot firmness significantly negatively correlated to time after operation (p<0.05, r=−0.89). The latter measure is often taken to be a measure of platelet function but here its decrease would appear to be due to increasing fibrinogenemia rather than weaning platelet function. *: significant correlation p<0.05. ADP: adenosine diphosphate. AUC: area under curve. MCF: maximum clot firmness. N/S: no significant correlation.

**Figure 2:**
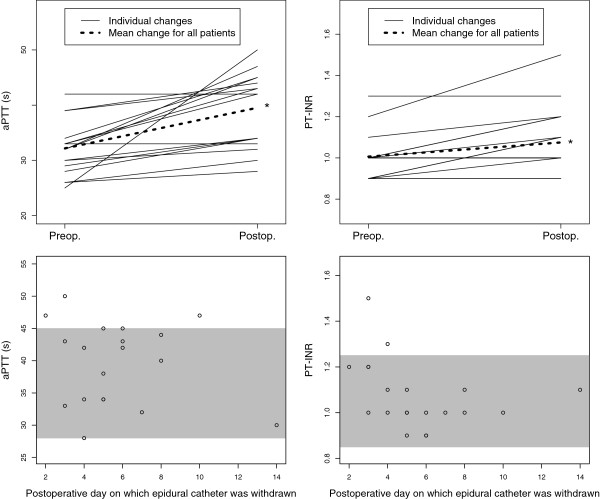
**Perioperative dynamics of activated partial thromboplastin time and prothrombin time-international normalized ratio.** These results give an overall impression of postoperative normo- or hypocoagulability. Shaded areas indicate the manufacturers’ reference ranges. aPTT: activated partial thromboplastin time. PT-INR: prothrombin time-international normalized ratio. Preop: preoperative test results. Postop: test results taken on the day on which epidural catheters were removed. s: seconds. *: significant difference between pre- and postoperative results (p<0.05, Student’s paired *t*-test).

Postoperative aPTT and PT-INR results were as expected significantly prolonged at the time of removal of epidural catheters in comparison to those taken preoperatively (aPTT: mean 39.5 seconds, SD 0.15 versus 32.2 seconds, SD 4.84; PT-INR: mean 1.08, SD 0.15 versus 1.01, SD 0.11; see Figures 1 and 2). There was also, unsurprisingly, a significant correlation (p<0.05; r=0.76) between the length of time after operation and Plc (see Figure 1). Normal values are shown in Table 1.

**Table 1:** Summary of assays performed

**Test**	**Vial**	**Apparatus**	**Place of analysis**	**Normal range**
ROTEM® using the following reagents: EXTEM, FIBTEM®, INTEM, APTEM, NATEM, HEPTEM®, recording the following results: CT (Clotting time), CFT (Clot formation time), Alpha-Angle (AA), MCF (Maximum Clot Firmness), ML (Maximum Lysis).	2.7mL citrate tubes for ROTEM® analysis (3.2% citrate, BD Vacutainer® Systems, Plymouth, UK)	ROTEM®, Pentapharm, Munich, Germany	Point-of-care laboratory, Intensive Care Unit	See Table 2.
Multiplate® using the following agonists: adenosine diphosphate, collagen, thrombin receptor activator, recording the following results for each agonist: area under curve (AUC), aggregation (AU), velocity (AU/min).	3.0mL Hirudin tubes. Dynabyte GmbH, Munich, Germany.	Multiplate®, Roche, Basel, Switzerland		See Table 2.
aPTT: activated partial thromboplastin time	2.7mL citrate tubes (3.2% citrate, BD Vacutainer® Systems, Plymouth, UK)	PTT-Automate, Stago (Asnière sur Seine, France)	Hospital’s usual clinical chemistry laboratory	28 to 45 seconds at the time of the study
PT-INR: prothrombin time-international normalized ratio		Stago prothrombin complex assay, Stago calibrated by Equalis, (Uppsala, Sweden)		≤1.2
Plc: platelet count	3.0mL K_2_EDTA tubes (BD Vacutainer® Systems, Plymouth UK)	Sysmex XE 5000 cell counter, Sysmex Corp., (Kobe, Japan).		165 to 387 million/mL and 145 to 387 million/mL for women and men respectively

### Multiplate® test results in the 20 patients

Median Multiplate® area under curve (AUC) was for all three tests within the reference ranges (see Table 2): after activation by adenosine diphosphate (ADP) it was 98U; after activation by collagen (COL) it was 104U and after activation with thrombin receptor activator (TRAP) it was 128U. AUC after activation with ADP correlated significantly to the length of time between operation and testing (see Figure 2) but AUC after activation with COL or TRAP did not (not shown).

**Table 2:** **Reference ranges for ROTEM® (Manufacturer’s information) and Multiplate®**[6]

**ROTEM®**	**CT (s)**	**CFT (s)**	**Alpha angle (°)**	**MCF (mm)**
EXTEM	38–79	34–159	63–83	50–72
APTEM	38–79	34–149	63–83	50–72
INTEM	100–240	30–110	70–83	50–72
HEPTEM®	100–240	30–110	70–83	50–72
NATEM	300–1000	150–700	30–70	40–65
FIBTEM®	-	-	-	9–25
(EXTEM-MCF)-(FIBTEM®-MCF)	-	-	-	41–48
**Multiplate®**	**AUC (U)**	**Aggr (AU)**	**Vel (AU/min)**	
ADPtest	57–113	108–122	16–19	
COLtest	72–125	126–140	18–21	
TRAPtest	84–128	140–152	24–26	

CT: Clotting time. s: seconds. CFT: Clot formation time. MCF: Maximum clot firmness. AUC: Area under curve. Aggr: aggregation. Vel: velocity. U: units. AU: area units. min: minute.

### ROTEM® test results in the 20 patients

Out of a total of 480 results, 18 (4%) from the 20 patients (24 parameters per patient) were not included: results were excluded in four patients due to desiccation artifacts, caused by the surface of the sample drying and clotting, or an inconsistent pattern of results suggesting that the wrong reagents had been used. Median results for each test’s results were within the normal ranges (see Table 2). Median maximum clot firmness (MCF) for each of the assays was as follows: EXTEM: 71mm; FIBTEM®: 30mm; INTEM: 68mm; HEPTEM®: 66mm; APTEM: 70mm; NATEM: 66mm. There were no significant differences between EXTEM and APTEM results, which would have indicated hyperfibrinolysis. Of the results, 61 (13%) indicated mild hypercoagulability and 16 of these were NATEM-clot formation time (CFT) measurements. Five results (1%) indicated hypocoagulability, but four of these were HEPTEM® results in patients whose INTEM results were normal, which ought to be impossible since HEPTEM® is identical to INTEM other than it contains heparinase, which would not be expected to inhibit coagulation. There was no significant difference between results from INTEM and HEPTEM®, which would have suggested an excessive heparin effect.

### Case studies of the four patients with abnormal routine coagulation results

#### Case report 1: normal ROTEM® and Multiplate® despite a prothrombin time-international normalized ratio of 1.5

A previously healthy 26-year-old woman weighing 67kg and not on any medication other than aluminum oxide and antacids due to dyspepsia, presented with a 2-month history of jaundice which later proved to be due to chronic pancreatitis. Since magnet resonance imaging suggested pancreatic malignancy, Whipple’s procedure was carried out, involving extensive pancreas resection. The operation was conducted under uncomplicated combined inhalational general and epidural anesthetic: the total perioperative hemorrhage was 700mL and in addition to crystalloid infusions (Ringer’s acetate and glucose solution), this patient received 2000mL of hydroxyethyl starch (Venofundin® 60mg/mL) and 250mL of 5% human albumin. Routine coagulation tests on the first postoperative day indicated a coagulopathy (see Table 3). Thrombosis prophylaxis was omitted that evening but continued thereafter. On the morning of the fourth postoperative day, ROTEM® and Multiplate® results were within their reference ranges despite a PT-INR of 1.5 (see Table 3). The patient’s epidural catheter was removed without complication 14 hours after the last dose of enoxaparin.

**Table 3:** Routine laboratory results for Case report 1

	**Hb g/L**	**PT-INR**	**APTT seconds**	**Plc 10**^**6**^**/mL**	**Creatinine umol/L**	**Albumin g/L**
Preoperative	123	1.2	33	266	51	38
First postoperative day	103	2.5*	44	152	43	28
At time of epidural catheter removal, fourth postoperative day.	107	1.5*	33	179	44	29

APTT: activated partial thromboplastin time. Hb: hemoglobin. Plc: platelet count. PT-INR: prothrombin time-international normalized ratio. *: outside normal reference range.

#### Case report 2: contamination by heparin demonstrated by ROTEM®

A 61-year-old man weighing 52kg but who had recently lost 50kg was admitted for resection of a lower esophageal tumor. He had previously received a subcutaneous venous port (Port-a-Cath™) for adjuvant chemotherapy and a percutaneous gastromy for nutrition. He had been prescribed oral esomeprazole, ondansetron, betamethasone, diazepam and mirtazapine in addition to transcutaneous fentanyl and subcutaneous ketobemidone. He received a combined general inhalational and epidural anesthetic and received 1000mL hydroxyethyl starch (Venofundin® 60mg/mL) during the operation. Blood loss during the operation was 300mL. It was considered clinically appropriate to remove his epidural catheter on the third postoperative day and coagulation tests were drawn from his heparinized Port-a-Cath™ (which has an internal volume of 1.3mL): the first 10mL blood was discarded. His aPTT was prolonged to 50 seconds and his Plc of 149 was slightly lower than expected.

ROTEM® results were all within the normal limits but there was a discrepancy between INTEM, a measure of the intrinsic pathway, and HEPTEM® which is identical other than it contains heparinase which removes any effect of heparin: clotting time (CT) was 100 seconds shorter for HEPTEM® than INTEM (154 seconds compared to 261 seconds). Multiplate® results were around the lower limits of the reference range (ADP-AUC was 60U). Routine tests the next day (see Table 4) sampled from a peripheral vein, were normalized despite the patient having received the same dose of low-molecular-weight heparin as on the previous days. The ROTEM® results show that the first sample taken was contaminated by heparin despite 10mL of dead space being withdrawn from the Port-a-Cath™ system which had an internal volume of 1.3mL. A repeat test could have being run on the same day, allowing earlier removal of this epidural catheter.

**Table 4:** Routine laboratory results for Case report 2

	**Hb g/L**	**PT-INR**	**APTT seconds**	**Plc 10**^**6**^**/mL**	**Creatinine umol/L**	**Albumin g/L**
Preoperative	142	1.0	25	230	49	36
On tenth postoperative day	107	1.0	50*	149	44	Not measured
At time of epidural catheter removal, 11th postoperative day.	113	1.0	31	178	45	Not measured

Summary of Case report 2’s routine laboratory studies. APTT: activated partial thromboplastin time. Hb: hemoglobin. Plc: platelet count. PT-INR: prothrombin time-international normalized ratio. *: outside normal reference range.

#### Case report 3: normal ROTEM® and Multiplate® results despite an activated partial thromboplastin time of 47 seconds and borderline prothrombin time-international normalized ratio at time of epidural removal

A previously healthy 52-year-old woman weighing 65kg received a combined general inhalational and epidural anesthetic for Whipple’s procedure due to pancreas cancer. Perioperative hemorrhage was 200mL and she received 1500mL hydroxyethyl starch (Venofundin® 60mg/mL) in addition to crystalloid infusions. She received standard thrombosis prophylaxis postoperatively. Her epidural catheter unfortunately failed to give effective analgesia and the decision to remove the catheter was made on the second postoperative day. Routine coagulation tests showed that PT-INR had increased to 1.2 and aPTT was slightly elevated to 47 seconds (see Table 5). ROTEM® and Multiplate® results were all normal: EXTEM-MCF was 70mm and Multiplate®-AUC was 90U. Her epidural catheter was removed without complication. As many clinicians would have delayed manipulation of this patient’s epidural given the slightly elevated aPTT and PT-INR at the upper end of normal, the ROTEM® and Multiplate® results might have contributed to the decision to withdraw the catheter without delay.

**Table 5:** Routine laboratory results for Case report 3

	**Hb g/L**	**PT-INR**	**APTT seconds**	**Plc 10**^**6**^**/mL**	**Creatinine umol/L**	**Albumin g/L**
Preoperative	130	1.0	32	201	60	Not measured
At time of epidural catheter removal, 2nd postoperative day.	123	1.2	47*	146	54	Not measured

Summary of Case report 3’s routine laboratory studies. APTT: activated partial thromboplastin time. Hb: hemoglobin. Plc: platelet count. PT-INR: prothrombin time-international normalized ratio. *: outside normal reference range.

#### Case report 4: hypercoagulant Multiplate® despite an activated partial thromboplastin time of 47 seconds at time of removal of epidural catheter

A previously healthy 49-year-old man weighing 93kg who had been a cigarette smoker for 30 years, and who took only omeprazole for chronic gastric regurgitation, presented with a 6-week history of weight loss and dysphagia which was diagnosed as a lower esophageal adenocarcinoma. He received a combined general inhalational and epidural anesthetic for esophageal resection and para-aortic lymph node dissection by laparotomy and thoracotomy: a total perioperative hemorrhage of 500mL was recorded and in addition to crystalloid infusions, this patient received 1500mL of hydroxyethyl starch (Venofundin® 60mg/mL) and 250mL of 5% human albumin. His postoperative recovery was uncomplicated and routine coagulation tests on postoperative day 8 were normal, although it may be noted that this patient’s blood albumin and hemoglobin were low (25g/L and 96g/L respectively). (See Table 6). Tests on postoperative day 10 showed a slightly elevated aPTT of 47 seconds, a thrombocytosis and Multiplate® results indicated strong platelet aggregation: the ADP test showed an AUC of 144U, AGG (aggregation) 244AU and VEL (velocity) 39.7AU/minute (see Table 2 for reference ranges). ROTEM® results were within the reference intervals and the epidural catheter was removed without complication. Again, ROTEM® and Multiplate® might have contributed to the decision to remove the epidural catheter despite an aPTT suggesting mild coagulopathy.

**Table 6:** Routine laboratory results for Case report 4

	**Hb g/L**	**PT-INR**	**APTT seconds**	**Plc 10**^**6**^**/mL**	**Creatinine umol/L**	**Albumin g/L**
Preoperative	148	Not measured		293	61	40
At time of epidural catheter removal, tenth postoperative day.	96	1.0	47*	524	71	25

Summary of Case report 4’s routine laboratory studies. APTT: activated partial thromboplastin time. Hb: hemoglobin. Plc: platelet count. PT-INR: prothrombin time-international normalized ratio. *: outside normal reference range.

## Discussion

This pilot study constitutes a small collection of somewhat heterogeneous data but does bring to light several important topics concerning ‘coagulative safety’ in the context of postoperative epidural anesthesia. There may be a place for these tests in routine practice, although this is currently not realistic due to these tests’ lack of validation in this setting, their operator-dependency and the fact that few hospital laboratories offer these tests for routine clinical use.

There appears to be a lack of concordance between whole blood viscoelastic tests and routine coagulation tests in the postoperative context, which brings current guidelines and the usefulness of both types of test in to question.

While PT-INR and aPTT would appear to indicate a trend towards postoperative coagulopathy in this and other studies [7], ROTEM® and Multiplate® suggested a trend towards postoperative hypercoagulation in this study.

Davignon *et al*. emphasize the importance of monitoring coagulation before removal of epidural catheters in case manipulation should disturb a clot and initiate an epidural hematoma, which they describe in a patient who received anticoagulation shortly after removal of an epidural catheter [8].

Current guidelines do not clearly describe what to do when there is a clinically pressing indication for removing an epidural catheter amidst laboratory tests indicating a coagulopathy: delaying removal of the epidural catheter in Case report 1 (in which PT-INR was 1.5) might possibly have delayed mobilization but there was no suspicion of local infection or sepsis, which would have made delayed withdrawal of the catheter potentially dangerous. The American Society of Regional Anesthesia and Pain Medicine recommends a PT-INR of 1.4 or lower but does not mention aPTT or Plc. That guidelines do not address aPTT is unfortunate since over half of the cases of spinal hematoma described by Miyazaki *et al*. were treated with anticoagulant therapy which would not necessarily be detected by the PT-INR alone [2]. Should anticoagulation be reversed, and if so how?

PT-INR is best validated for monitoring the effect of vitamin K antagonists such as warfarin, which was not something that we give patients undergoing major surgery. Viscoelastic tests are insensitive to increases in PT-INR: ROTEM®-CT is prolonged first when the PT-INR is around 3.5 (manufacturer’s information): neither TEG® nor ROTEM® are validated for reversal of vitamin K antagonism with prothrombin complex concentrate. Tissue factor can be used as an activator to give viscoelastic tests better sensitivity for PT, but this is at present not commercially available [9]. ROTEM® and Multiplate® nevertheless gave the clinician looking after the patient in Case report 1 the confidence to remove her epidural catheter [10].

There is a report by Hepner *et al*., of TEG®, technically similar to ROTEM®, being used to monitor coagulation at the time of removal of epidural catheters in 52 orthopedic patients treated with low-dose warfarin to give a PT-INR of up to 1.5 [11]. This is an attractive concept since the risk of spinal hematoma after epidural catheterization may be highest after such procedures and a point-of-care test might allow for more accurate prescription of warfarin [1]. TEG® was insensitive to warfarin’s effects in Hepner and colleague’s study which, like all studies in this area, was underpowered to draw any conclusion about TEG® and PT-INR’s predictive value regarding the risk of spinal hematoma. There are several commercial point-of-care whole-blood PT assays available: Hemochron Junior®, iStat® and Coaguchek Pro®.

There is no conclusive evidence that it is unsafe to place an epidural catheter when the Plc is less than 100×10^6^/mL, yet this is the generally accepted recommendation [4,12]. None of our patients had thrombocytopenia and it was of no surprise that both the number of platelets and Multiplate®-ADP-AUC increased with time after operation as part of the general postoperative inflammatory reaction. Being able to trust measures of platelet function in thrombocytopenic patients would be desirable, and being able to monitor the effect of attempted amelioration of platelet function with desmopressin for example, would be attractive since it might avoid unnecessary platelet transfusion.

Figure 1 shows three measures which correlate positively to length of time after operation, presumably as part of the inflammatory reaction. They are Plc, ROTEM®-EXTEM-MCF and Multiplate®-ADP-AUC. The difference between the MCF of ROTEM®-EXTEM and ROTEM®-FIBTEM® becomes smaller since the extrinsic pathway does not increase in activity as a whole despite hyperfibrinogenemia. ROTEM®-(EXTEM-FIBTEM®) would therefore not appear to be a useful measure of platelet function in the context of dynamic postoperative inflammation [13].

### Meticulous sampling technique is paramount

Case report 2 demonstrates that preanalytical errors such as contamination with heparin can remove any potential benefit that a test might offer. An experienced clinician, however, ought to notice a difference in CT of 100 seconds between HEPTEM® and INTEM, consider sampling technique and ask for a repeat blood sample taken by venepuncture or from a non-heparinized line. Since results from ROTEM® are available in real time, it should be possible to obtain these even before initial routine coagulation results are obtained from the hospital laboratory. ROTEM®-HEPTEM® and INTEM were certainly useful in this patient, who had several factors that predisposed to coagulation defects: malnutrition, multiple medications, a large infusion of hydroxyethyl starch, major operative trauma and slightly low Plc. It is noteworthy that viscoelastic tests are not capable of monitoring thromboprophylactic dosages of low-molecular-weight heparin [14].

It is significant that 18 of the 480 ROTEM® results (4%) were excluded due to artifacts or suspicion that the wrong reagents had been used. Point-of-care tests have the limitation that they are often used by clinicians who are competent to interpret the results but who are neither trained to use nor experienced in using the equipment. Running ROTEM® and Multiplate®, for example, involves pipetting several different reagents. The operator has ample opportunity to use the wrong or contaminated materials, or even the wrong blood sample. Some of these sources of error are eliminated by those hospital laboratories which have introduced ‘point-of-care’ tests with telemetry: samples are sent to the laboratory and run by trained and experienced technicians. Results are displayed in real time on a monitor at the Intensive Care Unit or operating theatres.

### Possible iatrogenic coagulopathy

It is troubling that patients 1, 3 and 4 had routine laboratory results suggesting a coagulopathy without our knowing for certain why. Lack of diagnosis precludes specific prevention and treatment. Patient 1’s spontaneously transient but somewhat dramatic increase in PT-INR from 1.2 to 2.5 has several possible explanations: dilutional coagulopathy and platelet inhibition by infusion of 2L of synthetic colloid and 250mL albumin; loss of coagulation factors by hemorrhage and possibly inability to synthetize new factors due to preoperative malnutrition and systemic inflammation [15]. It is also possible that our current practice of prescribing 40mg of enoxaparin as thrombosis prophylaxis to all patients regardless of weight leads to accumulation and coagulopathy in smaller patients who have decreased renal function. It is clearly of interest to prospectively and directly investigate coagulation factors and indicators of malnutrition in the perioperative period. We are currently running a study of this type.

### The importance of being sensible

Since there is no optimal or validated method to predict the risk of epidural hematoma one must be vigilant for signs and symptoms of epidural hematoma not only after catheterization but also after removing an epidural catheter. Magnetic resonance imaging should be carried out early to enable surgical intervention to avoid neurological damage.

So long as we are not sure why a patient’s coagulation tests indicate coagulopathy before removal of an epidural catheter, we cannot be sure how to treat them. Current strategies at our hospital include administration of between 10 and 30mg vitamin K per day and stopping enoxaparin, then taking new coagulation tests a day later. Transfusions of plasma have previously been given before the removal of catheters without further testing.

## Conclusions

This pilot study is new in that it examines ROTEM® and Multiplate® at the time of epidural catheter removal. These point-of-care tests may have a role to play in this setting since they showed a normal or hypercoagulative signal in most patients despite aPTT and PT-INR showing a trend towards the hypocoagulable.

Normal values need to be defined for viscoelastic tests and platelet aggregometry after major surgery. ROTEM®-(EXTEM minus FIBTEM®)-MCF does not appear to be a suitable measurement of platelet function.

At present we do not know enough about the pathophysiology of postoperative coagulation defects: the causes of prolonged PT-INR and aPTT should be investigated further, including the effects of synthetic starches.

## Methods

On the day on which patients’ epidural catheters were to be removed, venous blood was sampled from indwelling peripheral or central venous catheters, which is routine practice at our hospital. ROTEM® and Multiplate® were run on this blood at the same time as the standard coagulation tests described in Table 1. Routine test results, both pre- and postoperative, were retrieved from the hospital’s electronic notes system (Melior, Siemens Healthcare, Upplands Väsby, Sweden).

### ROTEM®

Thromboelastometry was carried out using the ROTEM® (rotational thromboelastometry) apparatus (Pentapharm, Munich, Germany) according to the manufacturer’s instructions. ROTEM® assays were run for 60 minutes.

Each sample was analyzed by ROTEM® using each of the six activators described in Table 7. Recalcification was carried out using 20μL of 0.2M calcium chloride (Star-TEM™). The following variables were registered: CT, CFT, alpha-angle (AA), and MCF.

**Table 7:** **Overview of ROTEM** ***® *** **parameters**

	**Trade name**	**Content**	**Action**	**Relevance**
1	NATEM (= ‘classic thromboelastometry’)	Only recalcification agent.	None – coagulation is activated by contact with the surface of the measurement container.	Produces a curve representing ‘whole blood coagulation’.
2	EXTEM	Tissue factor and phospholipids.	Activates the extrinsic pathway.	CT corresponds to PT. Curve represents clot formation, stability and fibrinolysis resulting from the extrinsic pathway.
3	INTEM	Ellagic acid and phospholipids.	Activates the intrinsic pathway.	CT corresponds to aPTT.
4	FIBTEM®	As EXTEM, plus cytochalasin D.	Cytochalasin D inhibits platelets.	Allows qualitative assessment of fibrinogen levels.
5	APTEM	As EXTEM, plus aprotinin.	Aprotinin inhibits plasmin and therefore fibrinolysis.	Comparing EXTEM and APTEM can rule in or out hyperfibrinolysis.
6	HEPTEM®	As INTEM, plus heparinase I.	Degrades heparin.	A comparison of HEPTEM® and INTEM indicates how much coagulation is affected by heparin.
**CT (Clotting time)**		Gives information about the kinetics of fibrin formation and clot development. Prolongation of CT may be a result of heparin or coagulation factor deficiency.		
**CFT (Clotting formation time)**		Gives information about the rate of clot formation. It is calculated as the interval between the onset of coagulation, arbitrarily defined as when the amplitude is 2mm, and the curve reaching an amplitude of 20mm. A prolonged CFT but normal MCF indicates a clot polymerization disorder.		
**AA (Alpha angle)**		Indicates the rate at which a solid clot forms. Both CFT and alpha angle are influenced by coagulation factors, platelet count and/or function and fXIII.		
**MCF (Maximum clot firmness)**		Shows the maximal strength and stability of the fibrin and platelet clot. A reduced MCF and normal CFT suggest lack of fibrinogen and/or platelets.		

Contents according to manufacturer’s information (Pentapharm, Munich, Germany).

### Multiplate®

Impedance aggregometry was carried out using the Multiplate® analyzer (Roche, Basel, Switzerland) according to the manufacturer’s instructions. Three platelet receptor agonists were applied: ADPtest, COLtest and TRAPtest, which activate coagulation with ADP, COL and thrombin receptor activating peptide 6 respectively. The following results were recorded for each agonist in each patient: area under curve, aggregation, and velocity. Normal values are described in Table 2.

Data was initially recorded on paper case report forms and later entered into an Excel sheet before statistical analysis using the statistical computing environment ‘R’ [16]. The significance of differences between pre- and postoperative test results were tested with Student’s paired *t*-test. Pearson’s product moment correlation test was used to define correlation coefficients.

## Consent

Written informed consent was obtained from all 20 patients involved in this study for publication of this case series and accompanying images. Copies of the written consents are available for review by the Editor-in-Chief of this journal. This study was approved by The Swedish Central Ethical Review Board (Lund, DNR 2010/482).

## Competing interests

The authors declare that they have no competing interests.

## Authors’ contributions

US and AG designed the study. AG collected the data. OT and US wrote the final manuscript, interpreted data and produced the figures and tables. US, OT, and AG were involved in producing an original manuscript which did not use case reports. All authors read and approved the final manuscript.
